# *Paraeutypella guizhouensis* gen. et sp. nov. and *Diatrypella longiasca* sp. nov. (Diatrypaceae) from China

**DOI:** 10.3897/BDJ.9.e63864

**Published:** 2021-03-26

**Authors:** Lakmali S. Dissanayake, Nalin N. Wijayawardene, Monika C. Dayarathne, Milan C. Samarakoon, Dong-Qin Dai, Kevin D. Hyde, Ji-Chuan Kang

**Affiliations:** 1 Engineering Research Centre of the Utilization for Characteristic Bio-Pharmaceutical Resources in Southwest, Ministry of Education, Guizhou University, Guiyang, Guizhou Province 550025, China Engineering Research Centre of the Utilization for Characteristic Bio-Pharmaceutical Resources in Southwest, Ministry of Education, Guizhou University Guiyang, Guizhou Province 550025 China; 2 Center for Yunnan Plateau Biological Resources Protection and Utilization, College of Biological Resource and Food Engineering, Qujing Normal University, Qujing, Yunnan 655011, China Center for Yunnan Plateau Biological Resources Protection and Utilization, College of Biological Resource and Food Engineering, Qujing Normal University Qujing, Yunnan 655011 China; 3 State Key Laboratory of Functions and Applications of Medicinal Plants, Guizhou Medical University, Guiyang 550014, China State Key Laboratory of Functions and Applications of Medicinal Plants, Guizhou Medical University Guiyang 550014 China; 4 Department of Plant Pathology, Agriculture College, Guizhou University, Guiyang, Guizhou Province, 550025, China Department of Plant Pathology, Agriculture College, Guizhou University Guiyang, Guizhou Province, 550025 China; 5 Center of Excellence in Fungal Research, Mae Fah Luang University, Chiang Rai, 57100, Thailand Center of Excellence in Fungal Research, Mae Fah Luang University Chiang Rai, 57100 Thailand

**Keywords:** *
Acer
*, morphology, novel taxa, phylogeny, Xylariales

## Abstract

**Background:**

In this study, we introduce a novel genus, *Paraeutypella*, of the family Diatrypaceae comprising three species viz. *Paraeutypella guizhouensis* sp. nov. and *P. citricola* (basionym: *Eutypella citricola*) and *P. vitis* (basionym: *Sphaeria vitis*). *Diatrypella longiasca* sp. nov. is also introduced, which forms a distinct clade in *Diatrypella* sensu stricto. The discovery of this new genus will contribute to expanding the knowledge and taxonomic framework of Diatrypaceae (Xylariales).

**New information:**

Generic delimitations in Diatrypaceae are unsettled because the phylogeny has yet to be resolved using extensive taxon sampling and sequencing of ex-type cultures. During an investigation of xylarialean fungi, we collected eutypella-like fungi which is distinct from *Eutypella* sensu stricto in our phylogenetic analyses (ITS and β-tubulin), thus, introduced as *Paraeutypella guizhouensis* gen. et sp. nov.. *Paraeutypella* is characterised by having 4–25 perithecia in a stroma each with 3–6 sulcate, long ostiolar necks. *Paraeutypella citricola* comb. nov. (basionym: *Eutypella citricola*) is introduced on *Acer* sp. from China. *Diatrypella longiasca* sp. nov. is introduced as a new species in *Diatrypella* sensu stricto. which has 2–5 ascomata per stroma and long ascospores, unusual when compared to other *Diatrypella* species and distinct phylogenetically.

## Introduction

Diatrypaceae Nitschke (Ascomycota, Xylariales) comprises 21 genera and more than 1,500 species (Senwanna 2017, Mehrabi et al. 2019, Dayarathne et al. 2020b, Wijayawardene et al. 2020). Species of this family are characterised by erumpent to immersed, rarely superficial, black or dark brown, eustromatic or pseudostromatic stromata and 8-spored or polysporous asci with hyaline to light brown, allantoid ascospores (Konta et al. 2020) in their sexual morph. Several asexual morph genera have been linked to the family Diatrypaceae, including *Cytosporina* Sacc. and *Libertella* Desm. (Glawe and Rogers 1984). *Cytosporina* Sacc. includes species with pycnidial and filiform conidia; *Libertella* Desm. includes species with acervula and filiform conidia (Glawe and Rogers 1984).

Members of Diatrypaceae are saprobes, pathogens or endophytes, associated with a wide range of hosts in terrestrial and aquatic environments (Mehrabi et al. 2019, Dayarathne et al. 2020a, Dayarathne et al. 2020b Konta et al. 2020). Dayarathne et al. (2020a), Dayarathne et al. (2020b) introduced two novel genera, *Halocryptosphaeria* Dayar. et al. and *Halocryptovalsa* Dayar. & K.D. Hyde from marine environments. Species of *Anthostoma* Nitschke, *Cryptosphaeria* Ces. & De Not., *Cryptovalsa* Ces. & De Not. ex Fuckel, *Diatrype* Fr., *Diatrypella* (Ces. & De Not.) De Not. and *Eutypella* (Nitschke) Sacc. have been reported as causal agents of canker diseases on a wide range of host plants worldwide (Hyde et al. 2020). The taxonomy and phylogeny of Diatrypaceae need to be resolved, as many genera are polyphyletic. Hence, fresh collections and sequences are required to define genera and establish their phylogenetic placement within the family.

*Diatrypella* was introduced by Cesati and De Notaris (1863) with *D. verruciformis* (Ehrh.) Nitschke as the type. The genus is characterised by conical to truncate, cushion-like or discoid stromata usually delimited by a black zone in host tissues, umbilicate or sulcate ostiolar necks, cylindrical, polysporous, long-stalked asci and allantoid, hyaline or yellowish ascospores in their sexual morph and a libertella-like coelomycetes asexual morph (Kirk et al. 2008, Hyde et al. 2020). Both *Cryptovalsa* and *Diatrypella* have polysporous asci and cannot easily be distinguished, based only on morphological comparisons (Acero et al. 2004, Vasilyeva and Stephenson 2005). Therefore, molecular data are essential for defining genera in Diatrypaceae (Mehrabi et al. 2015). There are 65 names of *Diatrypella* in Species Fungorum (2020) (http://www.indexfungorum.org/names/names.asp), but only 15 have molecular data in GenBank (Hyde et al. 2020).

In this study, we introduce a new genus, *Paraeutypella*, which shows eutypella-like morphology, but is distinct phylogenetically. *Paraeutypella* comprises three species viz. *Paraeutypella guizhouensis* sp. nov. and *P. citricola* (basionym: *Eutypella citricola*) and *P. vitis* (basionym: *Sphaeria vitis*). *Diatrypella longiasca* sp. nov. is also introduced, which forms a distinct clade in *Diatrypella* sensu stricto. Species novelties are confirmed by morphological comparisons along with micro-photographs and the phylogeny of combined ITS and β-tubulin sequence data.

## Materials and methods

### Sample collection and morphological observations

Dead twigs of *Acer palmatum* and undetermined plants were collected from China (Guiyang, Guizhou Province) during September to October 2019. Samples were observed with a stereomicroscope (SZX16, Olympus). Hand sections of the ascomata were mounted in distilled water and the following characters were measured: diameter and height of ascomata, width of the peridium, diameter and height of ostiolar necks, length and width of asci and ascospores. Melzer’s Reagent was used for testing the ascal apical ring reaction. Images were captured with a Canon EOS70D digital camera fitted to a compound microscope. Measurements were made with the Tarosoft (R) Image Frame Work programme and images used for figures processed with Adobe Photoshop CS6 software (Adobe Systems, USA). Single spore isolation was performed according to Chomnunti et al. (2014) and germinating spores were transferred to potato dextrose agar (PDA- Shanghai Bio-way Technology Co. Ltd.). The pure cultures were incubated at 18–20ºC for four weeks. The type specimens were deposited in the Cryptogamic Herbarium, Kunming Institute of Botany, Academia Sinica (HKAS), Chinese Academy of Science, Kunming and Chinese Academy of Science Herbarium (HMAS), Beijing, China. Ex-type cultures were deposited in the Kunming Institute of Botany Culture Collection (KUMCC). Facesoffungi and Index Fungorum numbers are provided as mentioned in Jayasiri et al. (2015) and Index Fungorum (http://www.indexfungorum.org) respectively.

### DNA extraction, PCR amplifications and sequencing

Fungal isolates were grown on PDA for 3–4 weeks at 25°C and total genomic DNA was extracted from 50 to 100 mg of axenic mycelium scraped from the edges of the growing cultures (Wu et al. 2001). EZgne^TM^ fungal gDNA extraction kit (BIOMIGA, Hangzhou City, Zhejiang Province, China) was used to extract DNA by following the manufacturer’s protocol. DNA extracts were stored at – 4°C for use in regular work and duplicated at –20°C for long term storage.

DNA sequence data were obtained from the internal transcribed spacer (ITS) and partial β-tubulin gene. ITS and β-tubulin were amplified by using the primers ITS5/ITS4 (White et al. 1990) and T1/T22 (O'Donnell and Cigelnik 1997), respectively. Polymerase chain reaction (PCR) was carried out in a volume of 25 μl, which contained 9.5 μl of ddH_2_O, 12.5 μl of 2× PCR Master Mix (2× Bench Top^TM^ Taq Master Mix, BIOMIGA, China), 1 μl of DNA template and 1 μl of forward and reverse primers (10 μM each) in each reaction. The PCR thermal cycle programme for all gene amplifications was as follows: initialisation at 95°C for 5 min, followed by 35 cycles of denaturation at 95°C for 30 s, annealing at 55°C for 50s, elongation at 72°C for 90s and final extension at 72°C for 10 min. Purification and sequencing of PCR products were done by Sangon Biotech, Shanghai, China.

### Molecular phylogenetic analyses

**Sequence alignment**

The sequence data generated in this study were analysed with closely-related taxa retrieved from GenBank (Table 1), based on BLASTn searches (https://www.ncbi.nlm.nih.gov) and recently published data (Mehrabi et al. 2019, Dayarathne et al. 2020b, Konta et al. 2020). ITS and β-tubulin were used for the analyses according to the previous studies listed above. Sequences (ITS and β-tubulin) were aligned using MAFFT v. 6.864b (Katoh et al. 2019) and manually improved when necessary in BioEdit v. 7.0 (Hall 1999). The single gene alignments were used to perform model test in MrModeltest 2.3 to estimate the best-fit evolutionary model under the Akaike Information Criterion (AIC) (Nylander 2004) and resulted in a GTR+I+G substitution model for each. Ambiguously aligned areas of each gene region were excluded and gaps were treated as missing data. Missing characters were assessed to be unordered and equally weighted.

**Phylogenetic Analyses**

Maximum Likelihood (ML) analysis was performed using RAxML-HPC2 on XSEDE (8.2.8) (Stamatakis 2014) in the CIPRES Science Gateway platform (Miller et al. 2010) using the GTR+I+G model of evolution. Bootstrap supports were obtained by running 1,000 pseudo-replicates. Bayesian analysis was conducted with MrBayes v. 3.1.2 (Huelsenbeck and Ronquist 2001) to evaluate Bayesian posterior probabilities (BYPP) (Rannala and Yang 1996, Zhaxybayeva and Gogarten 2002) by Markov Chain Monte Carlo sampling (BMCMC). GTR+I+G was used as the substitution model. Six simultaneous Markov chains were run for 2,000,000 generations and trees were sampled every 200^th^ generation. The distribution of log-likelihood scores was examined to determine the stationary phase for each search and to decide if extra runs were required to achieve convergence, using the programme Tracer 1.5. The first 10% of generated trees were discarded and remaining 90% of trees were used to calculate posterior probabilities of the majority rule consensus tree. All trees were visualised in FigTree v.1.4.0 (Rambaut 2012) and the final layout (Fig. 1) was done with Microsoft PowerPoint (2013). The final alignment and tree were registered in TreeBASE under the submission ID: 27435 (http://purl.org/phylo/treebase/phylows/study/TB2:S27435?x-access-code=3101b93c442e7aa253174d89df7a500c&format=html).

## Taxon treatments

### Diatrypella

(Ces. & De Not.) De Not. 1863

479B19E3-08E1-5292-8686-0C63963A71CC

Diatrypella Diatrypella verruciformis (Ehrh.) Nitschke(Fr.) Reason for typification: Indication or designation of a type in the protologue, names of genera or subdivisions of genera (Art. 10, 40).

#### Description

Notes – *Diatrypella* was introduced by Cesati & De Notaris (1863) and is typified as *Diatrypella verruciformis* (Ehrh.) Nitschke. There are 146 epithets listed in Index Fungorum (2020). This genus was established to accommodate members of stromatic Sphaeriales which were characterised by ovoid and numerous ascospores and we introduce a new species viz. *Diatrypella longiasca*, based on new collections from China.

### Diatrypella longiasca

L.S. Dissan., J.C. Kang & K.D. Hyde sp. nov.

BBACA31F-8319-58EB-8877-9575D0771481

IF557952

FoF09151

#### Materials

- **Type status:** Holotype. **Taxon:** kingdom: Fungi; phylum: Ascomycota; class: Sordariomycetes; order: Xylariales; family: Diatrypaceae; genus: Diatrypella; specificEpithet: longiasca; taxonRank: species; scientificNameAuthorship: L.S. Dissan., J.C. Kang & K.D. Hyde, sp. nov.; **Location:** country: China; stateProvince: Guizhou Province; county: Guiyang; locality: Guizhou University Garden (South); **Identification:** identifiedBy: L.S. Dissanayake; **Record Level:** institutionID: HMAS 290656; collectionID: HMAS 290658; institutionCode: Chinese Academy of Science, Kunming and Chinese Academy of Science Herbarium; collectionCode: Kunming Institute of Botany Culture Collection; datasetName: CLD 42
- **Type status:** Other material. **Record Level:** type: isotype; institutionID: HMAS 290658; collectionID: KUMCC 20-0022; institutionCode: Chinese Academy of Science, Kunming and Chinese Academy of Science Herbarium; collectionCode: Kunming Institute of Botany Culture Collection

#### Description

**Figure 2.**
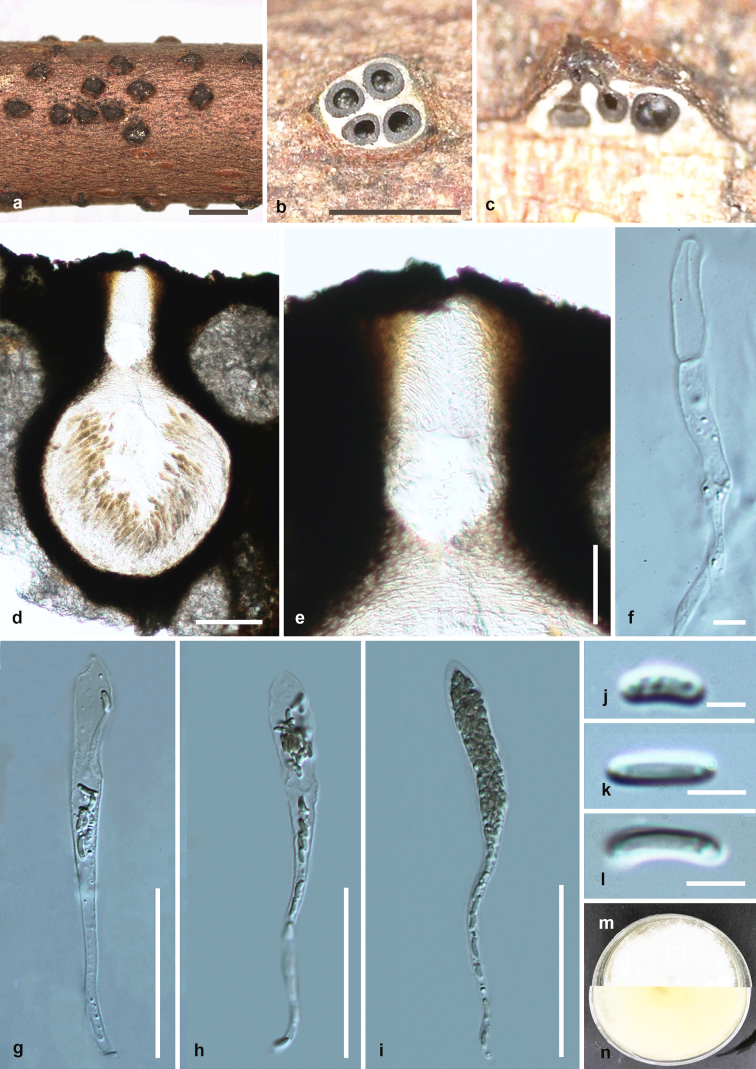
***Diatrypella longiasca*** (HMAS 290656, **holotype**) **a.** stromata on substrate; **b.** cross section of a stroma; **c, d.** vertical section through stroma showing ostiole and perithecia; **e.** ostiolar canal; **f.** paraphyses; **g–i.** asci; **j–l.** ascospores; **m, n.** culture on PDA from **m** above, **n** below after 6 weeks. Scale bars: 500 µm (**a, b**), 100 µm (**d**), 50 µm (**e, g–i**), 5 µm (**f, j–l**).

Saprobic on dead twigs (Fig. 2). **Sexual morph**: Stromata 0.5–0.7 mm in diam., well-developed, solitary to gregarious, erumpent, black, immersed, globose to subglobose. Ascomata 525–540 μm high, 470–510 μm diam. (x̄ = 532 × 490 μm, n = 15), perithecial, surrounded by white entostroma, immersed in stromata, 2–5 perithecia arranged in a valsoid configuration, subglobose, individual ostiole with a long neck. Neck 180–190 μm long (x̄ = 185 μm, n = 15), cylindrical, with periphyses. Peridium 36–45 μm wide (x̄ = 40.5 μm, n = 20), composed of two layers: outer layer of black, thick-walled cells; inner layer; hyaline, thick-walled cells forming textura angularis. Hamathecium 259–287 μm wide (x̄ = 273 μm, n = 20), composed of cells 3–5 μm wide (x̄ = 4 μm, n = 20), paraphyses arising from base of perithecia, hyaline, long, narrow, unbranched, septate, guttulate, narrowing and tapering towards apex. Asci 105–155 × 10–16 μm (x̄ = 130 × 14 μm, n = 30), polysporous, unitunicate, clavate, apically pointed, with a J-apical ring, long pedicellate (40–50 μm). Ascospores 4–8 × 1–2 μm (x̄ = 6 × 1.5 μm, n = 30), overlapping, hyaline, yellowish in mass, allantoid, aseptate, guttulate, smooth-walled. **Asexual morph**: Undetermined.

Culture characteristics – Colonies on PDA reaching 21 mm diam. after 2 weeks at 20–25^o^C, medium dense, circular to slightly irregular, slightly raised, cottony surface; colony from above: at first white, becoming buff; from below: yellowish white at margin, yellow to brown at centre; mycelium yellowish.

#### Etymology

The specific epithet *longiasca* refers to the long asci.

#### Notes

*Diatrypella longiasca* shares similar characters with *D. vulgaris* in having erumpent stromata through the bark often surrounded by remaining adherent epidermis or woody fragments and asci with many ascospores. However, *D. vulgaris* is different from *D. longiasca* in having longer ascospores (8–10 × 2–2.5 μm vs. 4–8 × 1–2 μm) (Trouillas et al. 2011). *Diatrypella vulgaris* has 4–8 ascomata per stromata, while *D. longiasca* comprises 2–5 ascomata per stromata. Comparison of the ITS 12% (73/570) and β-tubulin 13% (56/432) nucleotide differences, phylogenetic analyses and significant morphological differences indicate that *D. longiasca* and *D. vulgaris* are distinct taxa. Thus, *D. longiasca* is introduced as a new species in *Diatrypella*, based on its morphology coupled with high support values from the phylogenetic analysis (96% ML, 0.99 BYPP, Fig. 1). A key to species related to *Diatrypella longiasca* is given below.

### Paraeutypella

L.S. Dissan., J.C. Kang, Wijayaw. & K.D. Hyde gen. nov.

715327BD-C54E-5547-AA28-C19AFE297701

IF557954

FoF09231

Paraeutypella Paraeutypella guizhouensis L.S. Dissan., J.C. Kang & K.D. Hyde Status: new species described in this paper.

#### Description

Saprobic on dead twigs. **Sexual morph**: Stromata immersed in bark of dead branches, erumpent, solitary or aggregated. Ascomata with groups of 4–25 perithecia arranged in a valsoid configuration, surrounded by white, powdery entostroma, perithecial, black or brown, subglobose, clustered, immersed in stromata. Necks papillate, with an elongated ostiolar neck, central ostiolar canal filled with periphyses, 3–6 sulcate. Peridium composed of two layers of cells of textura angularis; inner layers cells hyaline or light brown, outer layers cells dark brown to black. Hamathecium composed of paraphyses arising from the base of perithecia, elongate, filiform, narrow, unbranched, septate, guttulate, narrowing and tapering towards apex. Asci 8-spored, unitunicate, thin-walled, clavate to cylindrical clavate or spindle-shaped, long pedicellate, apical rings J-. Ascospores overlapping biseriate, allantoid, slightly to moderately curved, allantoid, several oil droplets in each end, hyaline to light brown, sometimes yellow in mass, aseptate. **Asexual morph**: Coelomycetous. Conidiomata black, subconic, multiloculate, largely prosenchymatous, producing yellowish conidial masses. Conidiophores not recorded. Conidiogenous cells cylindrical, tapering, arising from pseudoparenchyma or interwoven hyphae, proliferating percurrently or sympodially, rarely both ways. Conidia hyaline, single-celled, slightly to moderately curved, with flattened bases, becoming guttulate (description of asexual morph adapted from Glawe and Jacobs 1987).

#### Etymology

With reference to the morphological resemblance to *Eutypella*.

#### Notes

*Paraeutypella* is introduced to accommodate three species viz. *P. guizhouensis* sp. nov., as well as *P. citricola* and *P. vitis*, two species previously placed in *Eutypella* sensu lato. *Paraeutypella* is typified by *P. guizhouensis*, which was collected from undetermined dead twigs. *Paraeutypella* can be distinguished from *Eutypella* species by stromata with perithecia in groups of 4–25 arranged in a valsoid configuration, 3–6 sulcate, long ostiolar necks, while stromata of *Eutypella* comprise groups of 20–70 perithecia having comparatively shorter ostiolar necks with sulcate or smooth ostiolar necks. Strains of both genera appear in distinct clades in a phylogeny based on ITS and Beta tubulin data (Fig. 1), thereby justifying the erection of the new genus *Paraeutypella.* However, sequence data are not available for the type of *P. citricola* and *P. vitis*. A co-elomycetous asexual morph has been recorded for *P. vitis* as *Eutypella vitis* in culture (Glawe and Jacobs 1987).

### Paraeutypella guizhouensis

L.S. Dissan., J.C. Kang & K.D. Hyde sp. nov.

838FFF41-7D15-5297-89F4-8E3CC09B751A

IF557953

FoF09148

#### Materials

- **Type status:** Holotype. **Taxon:** kingdom: Fungi; phylum: Ascomycota; class: Sordariomycetes; order: Xylariales; family: Diatrypaceae; genus: Paraeutypella; specificEpithet: guizhouensis; **Location:** country: China; stateProvince: Guizhou Province; county: Guiyang; locality: Guizhou University Garden (North); **Identification:** identifiedBy: L.S.Dissanayake; **Event:** habitat: *Saprobic* on dead twigs.; fieldNumber: CLD018; **Record Level:** type: Holotype; institutionID: HMAS 290654; collectionID: KUMCC 20–0016; institutionCode: Chinese Academy of Science, Kunming and Chinese Academy of Science Herbarium; collectionCode: Kunming Institute of Botany Culture Collection; datasetName: CLD018
- **Type status:** Other material. **Record Level:** type: isotype; institutionID: HKAS 290655; collectionID: KUMCC 20-0017; institutionCode: Chinese Academy of Science, Kunming and Chinese Academy of Science Herbarium; collectionCode: Kunming Institute of Botany Culture Collection

#### Description

**Figure 3.**
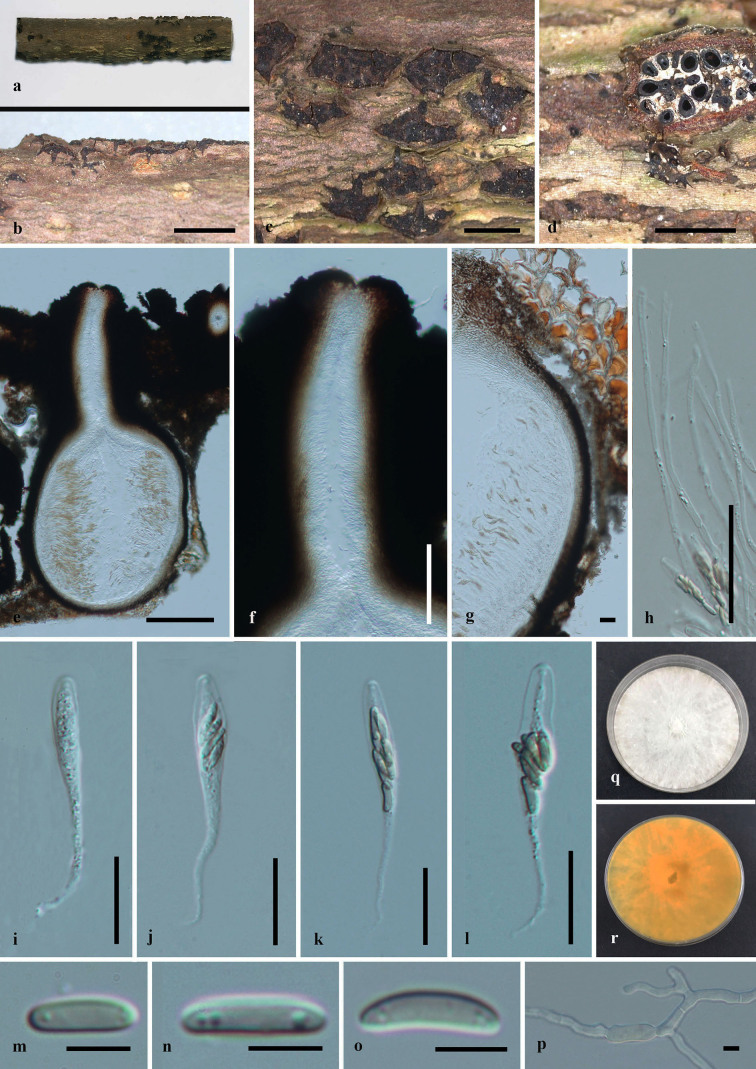
***Paraeutypella guizhouensis*** (HMAS 290654, **holotype**) **a–c.** stromata on substrate; **d.** cross section of a stromata; **e.** vertical section through an ascostroma showing ostioles and perithecia; **f.** ostiolar canal; **g.** peridium; **h.** paraphyses; **i–l.** asci; **m–o.** ascospores; **p.** germinating ascospore; **q, r.** cultures on PDA from above and below after 6 weeks. Scale bars: 500 µm (**b–d**), 200 µm (**e**), 100 µm (**f**), 20 µm (**g–l**), 5 µm (**m–p**).

Saprobic on dead twigs (Fig. 3). **Sexual morph**: Stromata immersed in bark of dead branches, erumpent, aggregated, circular to irregular, superficial, carbonaceous. Ascomata 590–600 × 470–480 μm (x̅ = 595 × 475 µm, n = 10), perithecial, with groups of 6–12 perithecia arranged in a valsoid configuration, subglobose, clustered, immersed in stromata, ostiolate. Neck 400–418 μm long (x̅ = 409 µm, n = 10), papillate, central ostiolar canal filled with periphyses, 3–4 sulcate. Peridium 22–35 μm wide, composed of two layers of textura angularis; inner layer cells light brown to hyaline, outer layers cells dark brown to black. Hamathecium hyaline. Paraphyses 1–2 μm wide (x̅ = 1.5 µm, n = 10), arising from base of perithecia, long, narrow, unbranched, septate, guttulate, narrowing and tapering towards apex. Asci 55–80 × 5–9 μm (x̅ = 67.5 × 7 μm, n = 20), 8-spored, unitunicate, thin-walled, clavate to cylindrical clavate, long pedicellate (25–30 μm), with a J- apical ring. Ascospores 7–11 × 1–3 μm (x̅ = 9 × 2 μm, n = 30), overlapping biseriate, allantoid, hyaline to light brown, smooth, aseptate, usually with 2–3 guttules. **Asexual morph**: Undetermined.

Culture characteristics – Colonies on PDA, reaching 21 mm diam. after 2 weeks at 20–25^o^C, medium dense, circular to slightly irregular, slightly raised, cottony surface; colony from above: at first white, becoming buff; from below: yellowish-white at margin, yellow to brown at centre; mycelium yellowish.

#### Etymology

The specific epithet *guizhouensis* refers to the locality in which the fungus was collected.

#### Notes

*Paraeutypella guizhouensis* resembles *P. vitis*, which comprises stromata that are erumpent through bark, with elongated perithecial necks and allantoid, slightly to moderately curved ascospores (Glawe and Jacobs 1987). However, *P. guizhouensis* differs from *P. vitis* in having comparatively longer ostiolar necks and longer asci (55–80 × 5–9 μm), while *P. vitis* has comparatively shorter ostiolar necks and shorter asci (40–46 × 6–8 μm) (Glawe and Jacobs 1987). *Paraeutypella vitis* (UCD2428TX) differs phylogenetically from our new taxon in 14% (80/576) base pairs in the ITS and 10% (42/405) base pairs in β-tubulin. Thus, *P. guizhouensis* is introduced as a new species in *Paraeutypella*, based on its morphology, base pair differences and phylogenetic analyses (94% ML, Fig. 1).

### Paraeutypella citricola

L.S. Dissan., Wijayaw., J.C. Kang & K.D. Hyde comb. nov.

28D3A0DD-067B-5B36-9EF5-4D66BC3371CB

IF558003

FoF09150

Paraeutypella citricola Speg., in Anales del Museo Nacional de Buenos Aires 6: 245, (1898)
= Eutypella citricola Syd. & P. Syd., Hedwigia 49: 80 (1909), nom. illegit., Art. 53.1

#### Materials

- **Type status:** Holotype. **Record Level:** institutionID: LPS-2120
- **Type status:** Paratype. **Occurrence:** recordedBy: Nalin N. Wijayawardene; **Taxon:** kingdom: Fungi; phylum: Ascomycota; class: Sordariomycetes; order: Xylariales; family: Diatrypaceae; genus: Paraeutypella; specificEpithet: citricola; **Location:** country: China; county: Guiyang; locationAccordingTo: Guizhou University Garden (South); **Identification:** identifiedBy: L.S.Dissanayake; **Event:** year: 2019; month: October; day: 5; habitat: on a dead branch of *Acer* sp.; **Record Level:** type: paratype; institutionID: HMAS 290660, HMAS 290659; collectionID: culture KUMCC 20–0024, KUMCC 20–0023; institutionCode: Chinese Academy of Science, Kunming and Chinese Academy of Science Herbarium; collectionCode: Kunming Institute of Botany Culture Collection

#### Description

**Figure 4.**
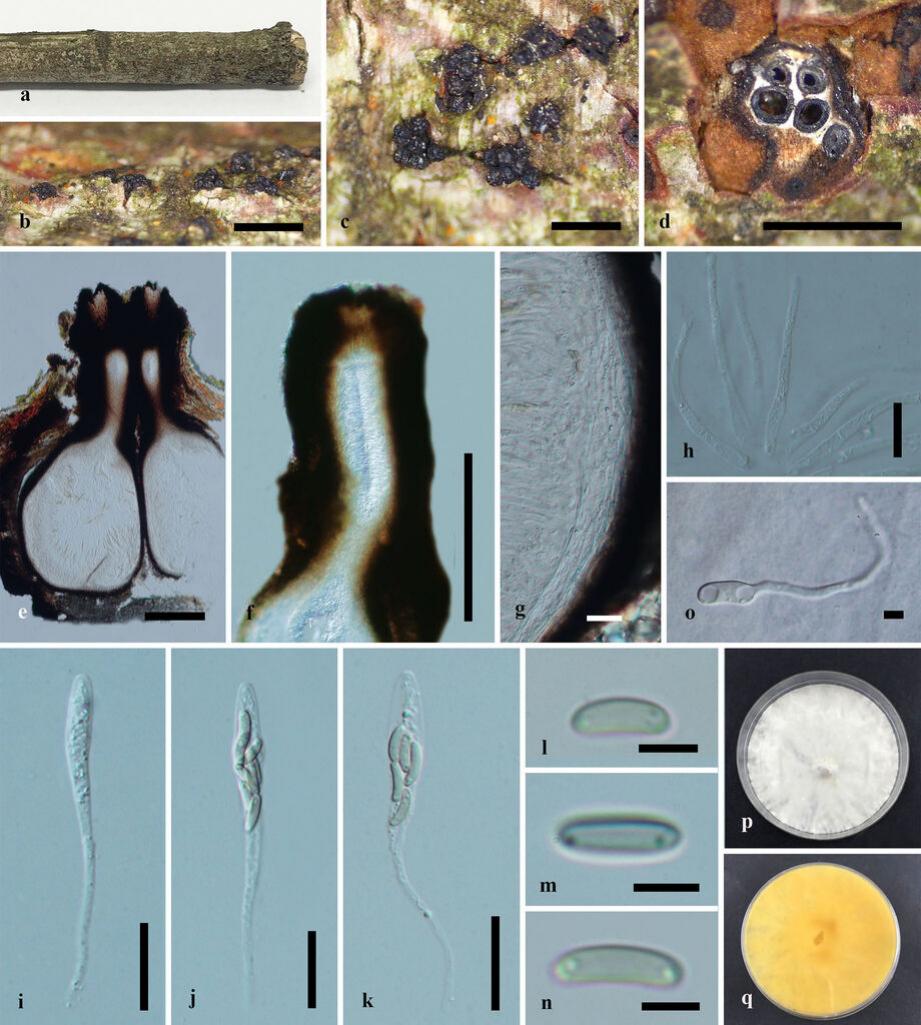
***Paraeutypella citricola*** (HMAS 290660) **a–c.** stromata on substrate; **d.** cross section of stroma; **e.** vertical section through stroma showing ostiolar necks and perithecia; **f.** ostiolar canal; **g.** peridium; **h.** paraphyses; **i–k.** asci; **l–n.** ascospores; **o.** germinating ascospore; **p, q.** culture on PDA after 6 weeks from above and below. Scale bars: 500 µm (**b–d**), 200 µm (**e–g**), 20 µm (**g**–**l**), 5 µm (**m–o**).

Saprobic on dead twigs of *Acer palmatum* (Fig. 4). **Sexual morph**: Stromata immersed in bark of dead branches, erumpent, solitary or aggregated, circular to irregular in shape, superficial, carbonaceous. Ascomata 410–430 × 430–470 μm (x̅ = 420 × 450 µm, n = 10), perithecial, with groups of 4–6 perithecia arranged in a valsoid configuration, black, subglobose, clustered, immersed in ascostroma with ostiolar neck. Necks 360–390 μm long (x̅ = 375 µm, n= 10), papillate, sulcate, central ostiolar canal filled with paraphyses. Peridium 27–40 μm wide, composed of two layers of textura angularis; inner layer cells hyaline, outer layer cells dark brown to black. Hamathecium composed of 3–7 μm wide (x̅ = 5 µm, n= 10), hyaline, paraphyses arising from base of perithecia, composed of long, narrow, unbranched, septate, guttulate, narrowing and apically truncate. Asci 70–75 × 5–8 μm (x̅ = 72.5 × 6.5 μm, n = 20), 8-spored, unitunicate, thin-walled, clavate to cylindrical clavate, long pedicellate (40–50 μm), J- apical ring. Ascospores 7–10 × 2–3 μm (x̅ = 8.5 × 2.5 μm, n = 30), overlapping biseriate, allantoid, hyaline to light brown, smooth, aseptate, usually with guttules. **Asexual morph**: Undetermined.

Culture characteristics – Colonies on PDA, reaching 21 mm diam. after 2 weeks at 20–25^o^C, medium dense, circular to slightly irregular, slightly raised, cottony surface; colony from above: at first white, becoming buff; from below: yellowish-white at margin, yellow to brown at centre; mycelium yellowish.

#### Notes

*Eutypella citricola* was described by Spegazzini (1898) from *Citrus* in Argentina and has since been reported to cause dieback on various woody plants in warm temperate and tropical regions (Farr and Rossman 2020). *Eutypella citricola* strains have previously been isolated from hosts such as *Citrus limon*, *C. sinensis*, *C. paradisi*, *Salix* spp., *Schinus molle*, *Ulmus procera* and *Vitis vinifera* (Trouillas et al. 2011, Mehrabi et al. 2016). In our study, we provide additional information for *P. citricola* from dead stems of *Acer* (Sapindaceae) in China. In morphology, our collection (HMAS 290660) resembles *Eutypella*, thus having pustulate stromata with stout, converging ostiolar necks and asci with eight spores. According to phylogenetic analysis, KUMCC 20–0024 closely groups with a collection of *E. citricola* (IRAN 2349C), which was collected on dead branches of *Salix* sp. (Salicaceae) in Gilan Province, Iran (Mehrabi et al. 2016) (Fig. 1). However, the IRAN 2349C strain is slightly different from our strain in having stromata with groups of 6–25 perithecia in a valsoid configuration and short ostiolar necks (100–300 µm), while our collection comprises stromata with groups of 4–6 perithecia in a valsoid configuration with a longer neck (356–385 μm). Based on phylogenetic analysis, both strains grouped in *Paraeutypella* sensu stricto (Fig. 1). Hence, the name *Eutypella citricola* is placed in *Paraeutypella* as *P. citricola*.

*Paraeutypella guizhouensis*, the type of *Paraeutypella*, morphologically resembles *P. citricola* both having immersed stromata, perithecia each with a long ostiolar neck and allantoid, aseptate ascospores with an oil droplet at each end. However, *Paraeutypella citricola* differs from *P. guizhouensis* by the number of perithecia within a stroma (4–6 vs. 6–12). A comparison of the ITS 1.0% (6/576) and β-tubulin 1.2% (5/406) between KUMCC 20-0024 and IRAN 2340C, ITS 1.0% (6/576) and β-tubulin 1.0% (5/406) between KUMCC 20-0024 and HVGRF01, HVVIT07 has been made.

### Paraeutypella vitis

L.S. Dissan., J.C. Kang & K.D. Hyde comb. nov.

54B1D003-FDD8-5A90-BBBC-6DF25F7AD89E

IF558004

FoF09426

Paraeutypella vitis Schwein., in Schr. Naturf. Ges. Leipzing 1: 39 (1822)
= Valsa vitis (Schwein.) Fuckel, Jb. Nassau. Ver. Naturk. 23-24: 199 (1870)
= Engizostoma vitis (Schwein.) Kuntze, Revis. Gen. pl. (Leipzig) 3 (3): 475 (1898)
= >Eutypella vitis (Schwein.) Ellis & Everh., The North American Pyrenomycetes: 490 (1892)

#### Notes

*Eutypella vitis* was collected from young shoots of grape vines in New York and was introduced by Ellis and Everhart (1982). According to our phylogenetic analyses, our new strain which represents *Eutypella vitis* (UCD 2291AR, USE2428TX) grouped as the sister clade (bootstrap support: 78% ML) to *Paraeutypella citricola* within *Paraeutypella* sensu stricto. Hence, in this study, we introduce the new combination, *Paraeutypella vitis*. *Paraeutypella vitis* shares similar morphologies to *Paraeutypella* species, such as having erumpent stromata through bark, 3–4 sulcate, long ostiolar necks, clavate asci, allantoid, slightly to moderately curved ascospores with several oil droplets in each end.

## Identification Keys

### Key to species similar to *Diatrypella longiasca*

**Table:** 

1	Ascospores 4–5 μm long on average	*Diatrypella major*
–	Ascospores 6–10 μm long on average	2
2	Entostroma yellowish or olive-green	3
–	Entostroma white	4
3	Asci larger, 120–150 × 15.5–21.5 μm	*D. tectonae*
–	Asci smaller, 40 × 8–12 μm	*D. frostii*
4	Stromata small, up to 2 mm diam.	5
–	Stromata larger than 2 mm	6
5	1–4 ascomata per stromata, on twigs of *Hevea brasiliensis*	*D. heveae*
–	3–4 ascomata per stromata, on seed pods of *Delonix regia*	*D. delonicis*
6	4–8 ascomata per stromata, (0.25–0.45 mm) with obscure ostiolar necks	*D. vulgaris*
–	2–5 ascomata per stromata, (0.5–0.7 mm) with prominent ostiolar necks	*D. longiasca*

### Key to species of *Paraeutypella*

**Table:** 

1	Stromata immersed	*Paraeutypella citricola*
–	stromata erumpent	2
2	Short ostiolar neck and longer asci (55–80 × 5–9 μm)	*P. vitis*
–	Long ostiolar neck and shorter asci (40–46 × 6–8 μm)	*P. guizhouensis*

## Analysis

**Phylogenetic analyses**

The combined ITS and β-tubulin matrix comprises 79 sequences that represents the genera in Diatrypaceae including the outgroup taxa. The best scoring RAxML tree is shown (Fig. 1) with a final ML optimisation likelihood value of -12709.069416. The matrix had 784 distinct alignment patterns, with 28.77% undetermined characters or gaps. Estimated base frequencies were: A = 0.226868, C = 0.263622, G = 0.232845, T = 0.27666; substitution rates AC = 1.218567, AG = 2.693651, AT = 1.272423, CG = 0.850048, CT = 3.427431, GT = 1.000000; proportion of invariable sites I = 0.100328; gamma distribution shape parameter α = 0.775027. All trees (ML and BYPP) were similar in topology and did not differ in generic relationships, which are in agreement with multi-gene phylogenies of previous studies.

Species of *Eutypella* are polyphyletic in our phylogram, while new isolates KUMCC 20-0023 and KUMCC 20-0024 grouped in a clade that comprises *Eutypella citricola* Syd. & P. Syd. and *Eutypella vitis* (Schwein.) Ellis & Everh. (Fig. 1). KUMCC 20-0016 and KUMCC 20-0017 formed a separate clade basal to *E. vitis* with high statistical support (94% ML) (Fig. 1). These species form a separate clade from the *Eutypella* clade. A novel genus is needed to accommodate these species, hence we introduce *Paraeutypella*.

Our new strains KUMCC 20-0021 and KUMCC 20-0022 are accommodated within *Diatrypella* with high statistical support (96% ML, 1.00 BYPP) as a distinct lineage.

## Discussion

This study introduces a new genus, *Paraeutypella* and accepts 22 genera in Diatypaceae. According to the previous analyses of combined ITS and β-tubulin sequence data, the genus *Eutypella* has been often identified as polyphyletic in Diatrypaceae (Trouillas et al. 2011, Mehrabi et al. 2016, Mehrabi et al. 2019, Dayarathne et al. 2016, Dayarathne et al. 2020a, Dayarathne et al. 2020b) and determined in our study as well (Fig. 1). The type of *Eutypella*, *E. cerviculata* (Fr.) Sacc. grouped with *E. semicircularis* S. Chacón & M. Piepenbr., *E. persica* Mehrabi et al. and *E. quercina* Mehrabi et al.

*Eutypella citricola* groups separately from *Eutypella* sensu stricto with *Eutypella vitis* and our newly-generated strains. These new strains are introduced as a new genus, *Paraeutypella* with three species viz. *P. citricola*, *P. guizhouensis* and *P. vitis*. We studied the morphological characteristics of the species belonging to this clade and found considerable morphological differences from *Eutypella* sensu stricto. The differences include stromata with 4–25 groups of perithecia in a valsoid configuration, 3–6 sulcate, long ostiolar necks; thus, we consider them to belong in a distinct genus from the *Eutypella* and hence, we introduce the novel *Paraeutypella*.

There does not appear to be any host-specificity since members of Diatypaceae are found on a wide range of hosts in various habitats. Diatypaceae species frequently have been identified as saprobes on the decaying wood of angiosperms. Few endophytes, such as *Diatrypella frostii* Peck and *Peroneutypa scoparia* (Schwein.) Carmarán & A.I. Romero, have been reported (de Errasti et al. 2010, Vieira et al. 2011, Grassi et al. 2014). Therefore, the family may have the potential for switching nutritional modes during the degradation of plant material (de Errasti et al. 2010, Grassi et al. 2014). Several species have been reported as pathogens, such as *Cryptosphaeria populina* (Pers.) Sacc., *C. pullmanensi* Glawe and *Eutypella parasitica* R.W. Davidson & R.C. Lorenz, causing canker disease (Glawe and Rogers 1984, Rappaz 1987, Ma et al. 2016), *Cryptovalsa ampelina* (Nitschke) Fuckel causing grapevine trunk disease (Luque et al. 2006), *Eutypa lata* (Pers.) Tul. & C. Tul. causing canker and dieback disease (Lardner et al. 2005) and *E. leptoplaca* (Durieu & Mont.) Rappaz contributing to the dieback of grapevines (Trouillas and Gubler 2004, Catal et al. 2007).

In our phylogenetic analyses, some species of *Diatrypella: D. favacea* (Fr.) Ces. & De Not., *D. iranensis* Mehrabi & Hemmati, *D. macrospora* Mehrabi et al. and *D. pulvinata* Nitschke formed a distinct lineage (Fig. 1) in *Diatrypella.* Similarly, some species of *Eutypella* (*E. caricae* (De Not.) Berl., *E. parasitica* R.W. Davidson & R.C. Lorenz and *E. virescens* Wehm.) often form distinct lineages within Diatrypaceae (Fig. 1). This may be due to lack of single-copy nuclear genes like β-tubulin or misidentified species.

## Supplementary Material

XML Treatment for Diatrypella

XML Treatment for Diatrypella longiasca

XML Treatment for Paraeutypella

XML Treatment for Paraeutypella guizhouensis

XML Treatment for Paraeutypella citricola

XML Treatment for Paraeutypella vitis

## Figures and Tables

**Figure 1a.**
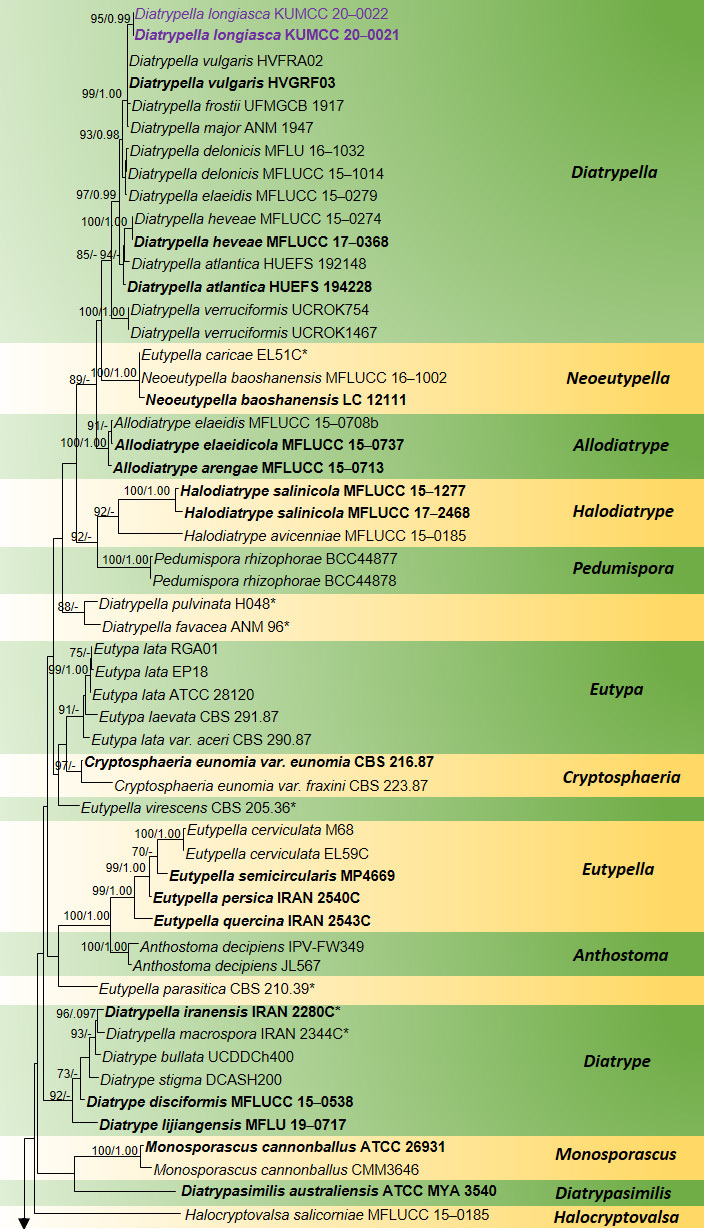


**Figure 1b.**
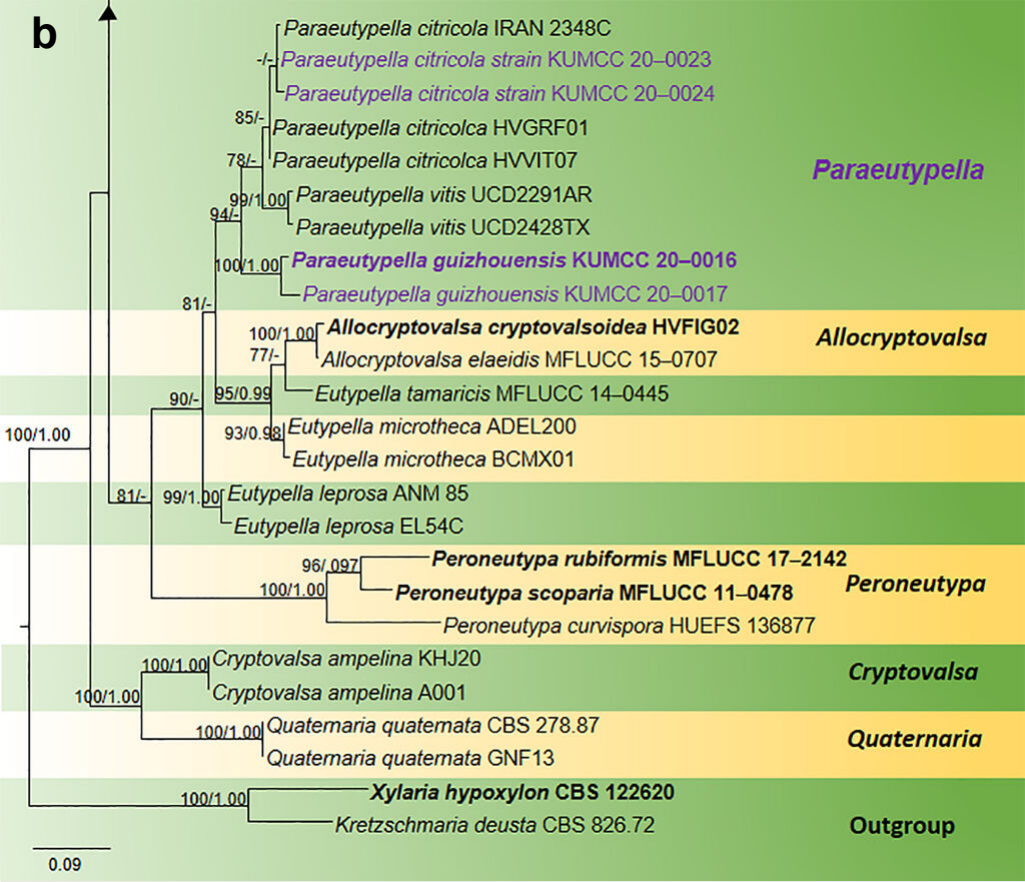


**Table 1.** Taxa used in the phylogenetic analysis and their corresponding GenBank accession numbers.

**Species**	**Strain no.**	**GenBank Accession no.**		**Reference**
		**ITS**	**β-tubulin**	
*Allocryptovalsa cryptovalsoidea* T	HVFIG02	HQ692573	HQ692524	Trouillas et al. (2011)
*A. elaeidis*	MFLUCC 15-0707	MN308410	MN340296	Konta et al. (2020)
*Allodiatrype arengae* T	MFLUCC 15-0713	MN308411	MN340297	Konta et al. (2020)
*A. elaeidicola* T	MFLUCC 15-0737	MN308415	MN340299	Konta et al. (2020)
*A. elaeidis*	MFLUCC 15-0708b	MN308413	NA	Konta et al. (2020)
*Anthostoma decipiens*	IPV-FW349	AM399021	AM920693	Nitschke (1867)
*A. decipiens*	JL567	JN975370	JN975407	Luque et al. (2012)
*Cryptosphaeria eunomia*	CBS 216.87	AJ302417	NA	Acero et al. (2004)
*C. var. eunomia*	CBS 223.87	AJ302421	NA	Acero et al. (2004)
*Cryptovalsa ampelina*	A001	GQ293901	GQ293972	Trouillas et al. (2015)
*C. ampelina*	KHJ20	KJ767718	KY352426	Mehrabi et al. (2015)
*Diatrypasimilis australiensis* T	ATCC MYA 3540	FJ430590	NA	Chalkley et al. (2010)
*Diatrype bullata*	UCDDCh400	DQ006946	DQ007002	Rolshausen et al. (2006)
*D. disciformis* T	MFLUCC 15-0538	KR092795	NA	Senanayake et al. (2015)
*D. lijiangensis* T	MFLU 19-0717	MK852582	MK852583	Thiyagaraja et al. (2019)
*D. stigma*	DCASH200	GQ293947	GQ294003	Trouillas et al. (2015)
*Diatrypella atlantica* T	HUEFS 194228	KM396615	KR363998	de Almeida et al. (2016)
*D. atlantica*	HUEFS 192148	KM396633	KT175563	de Almeida et al. (2016)
*D. delonicis* T	MFLUCC 15-1014	MH812994	MH847790	Hyde et al. (2019)
*D. delonicis*	MFLU 16-1032	MH812995	MH847791	Hyde et al. (2019)
*D. elaeidis* T	MFLUCC 15-0279	MN308417	MN340300	Konta et al. (2020)
*D. favacea*	ANM 96	KU320616	NA	de Almeida et al. (2016)
*D. frostii*	UFMGCB 1917	HQ377280	NA	Vieira et al. (2012)
*D. heveae* T	MFLUCC 17-0368	MF959501	MG334557	Senwanna (2017)
*D. heveae*	MFLUCC 15-0274	MN308418	MN340301	Konta et al. (2020)
*D. iranensis* T	IRAN 2280C	KM245033	KY352429	Mehrabi et al. (2015)
***D. longiasca*** T	**KUMCC 20-0021**	**MW039349**	**MW239658**	**This study**
***D. longiasca***	**KUMCC 20-0022**	**MW036141**	**MW239659**	**This study**
*D. macrospora* T	IRAN 2344C	KR605648	KY352430	Mehrabi et al. (2016)
*D. major*	ANM 1947	KU320613	NA	de Almeida et al. (2016)
*D. pulvinata*	H048	FR715523	FR715495	Pazoutova et al. (2012)
*D. verruciformis*	UCROK1467	JX144793	JX174093	Lynch et al. (2013), Luque et al. (2012)
*D. verruciformis*	UCROK754	JX144783	JX174083	Lynch et al. (2013)
*D. vulgaris*	HVFRA02	HQ692591	HQ692503	Trouillas et al. (2015)
*D. vulgaris* T	HVGRF03	HQ692590	HQ692502	Trouillas et al. (2015)
*Eutypa laevata*	CBS 291.87	AJ302449	NA	Acero et al. (2004)
*E. lata*	ATCC 28120	DQ006948	DQ006975	Rolshausen et al. (2006)
*E. lata*	EP18	HQ692611	HQ692501	Trouillas et al. (2011)
*E. lata*	RGA01	HQ692614	HQ692497	Trouillas et al. (2011)
*E. lata var. aceri*	CBS 290.87	HM164736	HM164770	Trouillas et al. (2010)
*Eutypella caricae*	EL51C	AJ302460	NA	Acero et al. (2004)
*E. cerviculata*	EL59C	AJ302468	NA	Acero et al. (2004)
*E. cerviculata*	M68	JF340269	NA	Arhipova et al. (2012)
*E. leprosa*	EL54C	AJ302463	NA	Acero et al. (2004)
*E. leprosa*	ANM 85	KU320622	NA	de Almeida et al. (2016)
*E. microtheca*	ADEL200	HQ692559	HQ692527	Trouillas et al. (2011)
*E. microtheca*	BCMX01	KC405563	KC405560	Paolinelli-Alfonso et al. (2015)
*E. parasitica*	CBS 210.39	MH855984	NA	Vu et al. (2019)
*E. persica* T	IRAN 2540C	KX828144	KY352451	Mehrabi et al. (2019)
*E. quercina* T	IRAN 2543C	KX828139	KY352449	Mehrabi et al. (2019)
*E. semicircularis* T	MP4669	JQ517314	NA	Chacón et al. (2013)
*E. tamaricis*	MFLUCC 14-0445	NA	KX453302	Thambugala et al. (2016)
*E. virescens*	CBS 205.36	MH855778	MH867286	Vu et al. (2019)
*Halocryptovalsa salicorniae*	MFLUCC 15-0185	MH304410	MH370274	Dayarathne et al. (2020b)
*Halodiatrype avicenniae*	MFLUCC 15-0948	MH304414	MH370278	Dayarathne et al. (2020b)
*H. salinicola* T	MFLUCC 15-1277	KX573915	KX573932	Dayarathne et al. (2016)
*H. salinicola*	MFLUCC17-2468	MN047113	NA	Dayarathne et al. (2016)
*Kretzschmaria deusta* T	CBS 826.72	KU683767	KU684190	U’Ren et al. 2016
*Monosporascus cannonballus* T	ATCC 26931	FJ430598	NA	Unpublished
*M. cannonballus*	CMM3646	JX971617	NA	Sales et al. (2010)
*Neoeutypella baoshanensis*	MFLUCC 16-1002	MT310662	NA	Phukhamsakda et al. (2020)
*N. baoshanensis* T	LC 12111	MH822887	MH822888	Hyde et al. (2019)
*Paraeutypella citricolca*	HVGRF01	HQ692579	HQ692512	Trouillas et al. (2011)
*P. citricola*	HVVIT07	HQ692589	HQ692521	Trouillas et al. (2011)
*P. citricola*	IRAN 2340C	KR605647	KY352439	Mehrabi et al. (2016)
***P. citricola***	**KUMCC 20-0023**	**MW040050**	**MW239663**	**This study**
***P. citricola***	**KUMCC 20-0024**	**MW040049**	**MW239662**	**This study**
***P. guizhouensis*** T	**KUMCC 20-0016**	**MW036142**	**MW239660**	**This study**
***P. guizhouensis***	**KUMCC 20-0017**	**MW039348**	**MW239661**	**This study**
*P. vitis*	UCD2291AR	HQ288224	HQ288303	Úrbez-torres et al. (2012)
*P. vitis*	UCD2428TX	FJ790851	GU294726	Úrbez-Torres and Gubler (2009)
*Pedumispora rhizophorae*	BCC44877	KJ888853	NA	Klaysuban et al. (2014)
*P. rhizophorae*	BCC44878	KJ888854	NA	Klaysuban et al. (2014)
*Peroneutypa curvispora*	HUEFS 136877	KM396641	NA	de Almeida et al. (2016)
*P. rubiformis* T	MFLUCC 17-2142	MG873477	NA	Shang et al. (2018)
*P. scoparia* T	MFLUCC 11-0478	KU940151	NA	Dai et al. (2016)
*Quaternaria quaternata*	CBS 278.87	AJ302469	NA	Acero et al. (2004)
*Q. quaternata*	GNF13	KR605645	KY352464	Mehrabi et al. (2016)
*Xylaria hypoxylon* T	CBS-122620	KY610407	KX271279	Peršoh et al. (2009)

**T**: Types strains, newly-generated sequences are indicated in bold, **NA**: No sequence available in GenBank, **ATCC**: American Type Culture Collection, Manassas, USA, **BCC**: BIOTEC Culture Collection, Bangkok, Thailand, **CBS**: Centra albureau voor Schimmel cultures, Utrecht, The Netherlands, **MFLU**: Mae Fah Luang University, Chiang Rai, Thailand, **CCMB**: Bahia Culture Collection of Microorganisms, **CMM**: Culture Collection of Phytopathogenic Fungi “Prof. Maria Menezes,” Federal Rural University of Pernambuco, Brazil, **MFLUCC**: Mae Fah Luang University Culture Collection, Chiang Rai, Thailand, **HKAS**: The Herbarium Mycologium, Institute of Microbiology Chinese Academy of Sciences, Beijing, China, **HUEFS**: Herbarium of the State University of Feira de Santana, **IRAN**: Iranian Fungal Culture Collection, Iranian Research Institute of Plant Protection, Tehran, Iran, **KUMCC**: Kunming Institute of Botany Culture Collection, Kunming, China.
